# Thymidylate synthase polymorphisms, folate and B-vitamin intake, and risk of colorectal adenoma

**DOI:** 10.1038/sj.bjc.6604056

**Published:** 2007-10-30

**Authors:** R A Hubner, J-F Liu, G S Sellick, R F A Logan, R S Houlston, K R Muir

**Affiliations:** 1 Section of Cancer Genetics, Institute of Cancer Research, 15 Cotswold Road, Sutton SM2 5NG, UK; 2 Division of Epidemiology and Public Health, Queen's Medical Centre, Medical School, University of Nottingham, Nottingham NG7 2UH, UK

**Keywords:** thymidylate synthase, polymorphism, folate, colorectal adenoma

## Abstract

The effects of polymorphisms in genes coding for key folate metabolism enzymes such as thymidylate synthetase (TS) on colorectal neoplasia risk are likely to be influenced by gene–gene and gene–nutrient interactions. We investigated the combined effects of three polymorphisms in the *TS* gene region, *TSER*, *TS* 3R G>C, and *TS* 1494del6, dietary intakes of folate and other B vitamins, and genotype for other folate metabolism variants, in a colorectal adenoma (CRA) case–control study. Individuals homozygous for *TS* 1494del6 del/del were at significantly reduced CRA risk compared to those with either ins/del or ins/ins genotypes (odds ratio 0.52; 95% confidence interval: 0.31–0.85, *P*=0.009). We also observed evidence of interactions between *TS* 1494del6 genotype and intake of folate, and vitamins B_6_ and B_12_, and *MTHFR* C677T genotype, with the reduction in risk in del/del homozygotes being largely confined to individuals with high nutrient intakes and *MTHFR* 677CC genotype (*P*_interaction_=0.01, 0.006, 0.03, and 0.07, respectively). *TSER* genotype, when considered either alone or in combination with *TS* 3R G>C genotype, did not significantly influence CRA risk. These findings support a role for TS in colorectal carcinogenesis, and provide further evidence that functional polymorphisms in folate metabolism genes act as low-risk alleles for colorectal neoplasia and participate in complex gene–gene and gene–nutrient interactions.

Folate coenzymes, acting as donors and acceptors of one-carbon units, play important roles in both DNA methylation and DNA synthesis, aberrations of which are known to play a major role in colorectal carcinogenesis (Choi and Mason, 2002). Functional polymorphisms in the genes coding for folate metabolism enzymes have the potential to influence either or both of these processes, making them attractive candidates as low-risk alleles for colorectal neoplasia (Sharp and Little, 2004). The relationship between polymorphism genotype and colorectal neoplasia risk is likely to be influenced by dietary factors that also alter enzyme function or availability of one-carbon units; thus, simultaneous consideration of both genotype and relevant dietary exposures is highly desirable when investigating disease risk (Kim, 1999).

Thymidylate synthetase (TS) is a key enzyme in folate metabolism catalysing the conversion of deoxyuridine monophosphate to deoxythymidine monophosphate, providing the sole *de novo* source of thymidine required for DNA synthesis and repair (Choi and Mason, 2000) (Figure 1). The TS substrate, 5,10-methylenetetrahydrofolate (5,10-methyleneTHF), is also a substrate for the methylenetetrahydrofolate reductase (MTHFR) enzyme in the production of 5-methylTHF, which is in turn converted into methionine required for DNA methylation. Thus, the reaction catalysed by TS alters the balance of folate metabolism away from DNA methylation and towards DNA synthesis.

Three polymorphisms mapping to the *TS* gene region, *TSER*, *TSER* 3R G>C, and *TS* 1494del6, alter either TS expression or TS mRNA stability (Horie *et al*, 1995; Ulrich *et al*, 2000; Pullarkat *et al*, 2001; Kawakami and Watanabe, 2003; Mandola *et al*, 2003, 2004), and have been reported in some studies to influence colorectal neoplasia risk when investigated individually (Ulrich *et al*, 2002, 2005; Chen *et al*, 2003). To date, no study has reported on the combined influence of all three polymorphisms, and we sought to address this by genotyping individuals in a large colorectal adenoma (CRA) case–control study including 673 cases and 301 controls. Other folate metabolism polymorphisms, in particular *MTHFR* C677T, may impact on the relationship between *TS* genotype and CRA risk (Ulrich *et al*, 2002); therefore, study participants were also genotyped for this and other putatively functional folate metabolism variants. Detailed data on folate and other B-vitamin intakes were also collected, permitting examination of potential gene–nutrient interactions.

Our principle prior hypothesis, based in part on results from studies of colorectal cancer (CRC) risk, was that *TS* genotypes associated with reduced enzyme function would be associated with reduced CRA risk. Due to their shared substrate, 5,10-methyleneTHF, we also anticipated gene–gene interactions between the *TS* and *MTHFR* variants. Gene–nutrient interactions were anticipated since B vitamins are cofactors for folate metabolism enzymes.

## MATERIALS AND METHODS

### Study population

Cases were recruited from two sources between 2001 and 2005. Five hundred and sixty-six cases were recruited from the UKCAP (United Kingdom Colorectal Adenoma Prevention) trial, a recently completed multicentre randomised trial of aspirin and folate for the prevention of CRA recurrence (Logan *et al*, 2006). A further 107 cases were recruited from colonoscopy clinics in the Midlands region of the United Kingdom. Regular aspirin or nonsteroidal anti-inflammatory drug (NSAID) use was an exclusion criteria for entry into the UKCAP trial. Patients excluded from UKCAP because of regular aspirin or NSAID use were not included in our study. The 107 additional CRA cases in our study were ascertained after recruitment to UKCAP was completed, and were therefore not considered for entry into the UKCAP study, thus, aspirin or NSAID use was not an exclusion criteria for these participants. All 673 cases had one or more histologically confirmed CRA⩾0.5 cm in size removed at full colonoscopy in the 6 months prior to enrolment. Individuals found to be free of adenoma on full colonoscopy at clinics in the Midlands region, and without a previous history of CRA or CRC, were recruited as controls. Three hundred and one controls were recruited between 2003 and 2005. Indications for colonoscopy included rectal bleeding or anaemia, altered bowel habit, abdominal symptoms, a family history of CRC, and asymptomatic screening (Table 1). All cases and controls were of Caucasian ethnicity.

Informed consent for the study was obtained from all participants, and the study was carried out with the Ethical Review Board approval in accordance with the tenets of the Declaration of Helsinki.

### Questionnaire data

Face-to-face interviews were conducted in the subjects' own home by dedicated research staff to gather information on lifestyle and medical factors, including ethnicity, demographic information, past medical history, detailed family history data, aspirin and NSAID use, multivitamin and individual vitamin supplement use, alcohol intake, smoking, and anthropometric measurements. For alcohol intake and smoking, subjects were asked about their average weekly consumption of beers, wine, and spirits, separately, or number of cigarettes smoked per day, in 10-year time intervals from the age of 10 years. From this information, the mean number of units of alcohol consumed per week in the 10-year time period prior to recruitment, and total number of pack years of cigarette smoking were calculated. All subjects also completed a food-frequency questionnaire (FFQ), which addressed their dietary intake in the 5 years prior to recruitment. To remove patients whose diet was inadequately captured by the FFQ, and incorrectly completed FFQs, patients with the highest and lowest 2.5% of energy intakes, or questionnaires with more than 10 empty data lines were excluded, leaving 583 cases and 277 controls in the dietary analyses. Nutrient intakes were determined using a nutrient conversion database, and adjusted for total energy intake using the linear residual regression method of Willett and Stampfer (Willett and Stampfer, 1986). Nutrient intake from supplements was not energy adjusted.

For cases recruited from the UKCAP study, both lifestyle and FFQs were completed at the time of entry to the UKCAP study, while for the remaining cases and all controls, questionnaires were completed at the time of recruitment to this study.

### Polymorphism selection

Candidate folate metabolism polymorphisms, other than the *TSER*, *TSER* 3R G>C, and *TS* 1494del6 variants were selected for genotyping on the basis of *a priori* evidence for functional effects on expressed proteins, and an influence on colorectal neoplasia risk. Database and literature searches were performed to establish a hierarchy of polymorphisms most likely to influence CRA risk, either directly or through interactions with folate metabolism-related dietary factors. The polymorphisms analysed were *MTHFR* C677T, *MTHFR* A1298C, *MTR* A2756G, and *MTRR* A66G.

### Genotyping

Constitutional DNA was extracted from EDTA venous blood samples using a standard salt extraction procedure, and quantified by PicoGreen (Invitrogen, Paisley, UK). The *TSER* and *TS* 1494del6 polymorphisms were analysed using fluorescent size discrimination on an ABI 3100 genetic analyser (Applied Biosystems, Foster City, CA, USA). A previously described PCR–RFLP assay was used to generate the *TSER* 3R G>C polymorphism genotypes (Mandola *et al*, 2003). Briefly, amplified fragments were digested with *Hae*III restriction enzyme (New England Biolabs, Hitchin, UK), and the digestion products separated by electrophoresis on a 4% agarose gel. *Hae*III digestion of the 3RG fragment produced bands of 11-, 27-, 28-, 44-, 47-, and 66-bp, while digestion of the 3RC fragment produced bands of 11-, 27-, 44-, 47-, and 94-bp. *TSER* genotypes were then divided into high (3RG/3RG, 3RG/3RC, and 3RG/2R) and low (3RC/3RC, 3RC/2R, and 2R/2R) TS expression groups (Mandola *et al*, 2003). *MTHFR* C677T, *MTHFR* A1298C, *MTR* A2756G, and *MTRR* A66G genotypes were generated using Taqman technology implemented on an ABI 7900HT sequence detection system (Applied Biosystems).

Genotyper (version 3.7) and ABI Prism 7900HT Sequence Detection System (version 2.1) software (Applied Biosystems) were used for fluorescent size discrimination and Taqman genotyping analyses, respectively. Genotyping assays for each polymorphism were validated using control samples of known homozygote wild-type, heterozygote, and homozygote variant genotype generated by direct sequencing. Unblinded control samples were included on each sample plate, and were correctly genotyped on 100% of occasions. Positive call rates for Taqman and Genescan analyses were 99 and 98%, respectively. Details of all PCR primer sequences and reaction conditions are available on request.

### Statistical analysis

The *χ*^2^ and *t*-tests were used to compare baseline characteristics between cases and controls, and to test genotype frequencies in the control group for evidence of departure from Hardy–Weinberg equilibrium. The relationship between genotype and risk of CRA was assessed by means of odds ratios (ORs) and 95% confidence intervals (CIs) calculated using unconditional logistic regression. Both unadjusted and adjusted ORs were calculated. Variables for sex, smoking, alcohol intake, and dietary intakes of fibre, folate, and vitamins B_2_ and B_6_ (from food and supplements combined) were included in adjusted analyses as these variables were found to significantly influence CRA risk. Aspirin or NSAID use was not included in multivariate analyses as it was an exclusion criterion for recruitment into the aspirin intervention trial from which the majority of cases were drawn. All adjustment variables were included in the model as continuous variables, apart from sex and alcohol intake, which was included as sex-specific tertiles. The associations between genotype and CRA risk were initially evaluated in the entire population, and subsequently separately in men and women. Further subsets of the population were based on polyp characteristics.

We then considered genotypes in combination with intakes of alcohol, and dietary folate and vitamins B_2_, B_6_, and B_12_. Energy-adjusted tertile cut points were used to designate low (lowest tertile), medium (middle tertile), and high (highest tertile) dietary intakes based on the distribution in control subjects. Alcohol intake tertiles were calculated for men and women separately, and tertiles for micronutrients were calculated for food-only intake, and for intake from food and supplements combined. Effect modification was evaluated by stratification on the variable of interest, and ORs within each stratum were compared. To test for linear trend, the tertile of nutrient intake was included as a continuous variable in the logistic regression model in which each tertile was assigned its median value. Interactions between variables with respect to CRA risk were explored using likelihood ratio testing comparing models with and without a multiplicative term for the two variables.

We also performed haplotype analysis for the *TSER* and *TS* 1494del6 variants and the *MTHFR* C677T and A1298C variants using genotype data from all subjects to infer chromosomal phase. Haplotypes were analysed using PHASE software, which implements the expectation-maximisation algorithm to estimate haplotype frequencies and individual haplotypes (Stephens *et al*, 2001). Possible haplotypes were tested for association with CRA risk by taking the most likely pair of haplotypes for each subject.

Statistical analyses were undertaken using STATA, version 8.2 (Stata Corporation, College Station, TX, USA), with the exception of the haplotype analyses above. All tests were two sided, and a *P*-value less than 0.05 was considered significant.

## RESULTS

Characteristics of the study population are shown in Table 1. Cases were more likely to be men and smokers than controls, and had a higher intake of alcohol. There were no significant differences between cases and controls in terms of age, family history of CRC, and body mass index. Energy adjusted dietary fibre intake was higher in controls, as were intakes of folate, and vitamins B_2_ and B_6_ when either intake from food only or intake from food and supplements combined was considered.

Allele frequencies for all polymorphisms were similar to previous reports in Caucasian populations, and all genotype frequencies in control subjects were in Hardy–Weinberg equilibrium (Le Marchand *et al*, 2002; Ulrich *et al*, 2002; Ogino and Wilson, 2003; Goode *et al*, 2004). We observed *TSER* repeat polymorphism alleles with four and six repeats (allele frequencies 0.2 and 0.1%, respectively) as has previously been reported (Marsh *et al*, 2000), and since the functional significance of such alleles is uncertain, subjects carrying these alleles were excluded from *TSER* association analyses. We also observed a *TSER* two-repeat allele with a C base at position 12 of the first repeat with a frequency of 0.5%, confirming a recent report of the existence of a *TSER* 2RC allele (Gusella *et al*, 2006).

Both *TS* 1494del6 and *MTHFR* C677T genotypes influenced CRA risk (Table 2). Individuals homozygous for the *TS* 1494del6 deletion were at 50% reduced risk compared to homozygous wild-type or heterozygous individuals combined (OR 0.52; 95% CI: 0.31–0.85, *P*=0.009), and the inverse association was more apparent when the analysis was restricted to cases whose largest adenoma was located in the colon rather than the rectum (OR 0.40; 95% CI: 0.23–0.71 and OR 0.87; 95% CI: 0.45–1.68, respectively). Individuals with homozygous *MTHFR* 677TT genotype were at significantly increased CRA risk compared to wild-type individuals and heterozygotes combined (OR 1.71; 95% CI: 1.03–2.86, *P*=0.04), and this influence was more apparent in men than women (OR 2.27; 95% CI: 0.90–5.73 and OR 1.41; 95% CI: 0.74–2.66, respectively), and when the analyses were restricted to cases whose largest adenoma was located in the rectum (OR 2.40; 95% CI: 1.24–4.62). Adjustment for *MTHFR* C677T polymorphism genotype did not significantly alter the association between *TS* 1494del6 and adenoma risk; however, when stratified by both *TS* 1494del6 and *MTHFR* C677T genotypes, the reduced risk associated with the del/del genotype was confined to individuals with CC genotype (OR 0.31; 95% CI; 0.15–0.63 for CC genotype individuals, and OR 1.02; 95% CI: 0.43–2.40 for CT/TT genotypes, *P*_interaction_=0.07). When the two variants were considered in combination, an over threefold increased risk (OR 3.68; 95% CI: 1.67–8.10, *P*=0.001) was observed in individuals with ‘high-risk’ genotypes for both variants (ins/ins or ins/del for the *TS* 1494del6 variant and *MTHFR* TT homozygotes) compared to individuals with ‘low-risk’ genotypes (del/del and CC or CT). When the *TS* 1494ins and *MTHFR* 677T alleles were considered ‘high-risk alleles’, and the number of high-risk alleles carried was included as a continuous variable in the analysis, then each additional high-risk allele was associated with a 20% (95% CI: 3–40%, *P*_trend_=0.02) incremental increase in risk.

We also observed significant interactions between *TS* 1494del6 genotype and dietary food intakes of folate, and vitamins B_6_ and B_12_ (Table 3). Among individuals with folate intake ⩾383 *μ*g day^−1^ (highest tertile), those with del/del genotype were at markedly reduced CRA risk compared to those with ins/ins genotype, whereas in individuals with folate intake below 383 *μ*g day^−1^ (low/medium tertile), the del/del genotype was associated with only a modest risk reduction. Similar interactions between *TS* 1494del6 genotype and vitamin B_6_ and B_12_ intakes were observed with the risk reduction associated with the del/del genotype consistently being confined to individuals with high nutrient intakes. Adjusting for *MTHFR* C677T genotype did not modify these gene–nutrient interactions, and similar interactions were observed if nutrient intake from food and supplements combined was considered (*P*_interaction_=0.07, 0.006, and 0.02, for folate, and vitamin B_6_ and B_12_ intakes, respectively). Furthermore, these gene–nutrient interactions were robust to other methods of classifying nutrient intake such as above and below median intake, with the lowest risks consistently occurring in individuals with high-nutrient intakes and del/del genotype. The *P*_int_-values for the interactions between *TS* 1494del6 genotype and nutrient intakes of folate, and vitamins B_6_ and B_12_ from food only categorised into two groups (high and low based on the median values in control subjects, folate 340 *μ*g day^−1^, vitamin B_6_ 2.42 mg day^−1^, and vitamin B_12_ 6.32 *μ*g day^−1^) were 0.18, 0.04, and 0.12, respectively. The corresponding values for nutrient intakes from food and supplements combined were 0.08, 0.01, and 0.09, respectively (corresponding median values 372 *μ*g day^−1^, 2.58 mg day^−1^, and 6.59 *μ*g day^−1^).

We also observed evidence of interactions between folate and alcohol intake and *MTHFR* C677T genotype, although nonsignificantly, with the highest adenoma risks occurring in individuals with the *MTHFR* 677TT genotype and low/medium folate or high/medium alcohol intakes. Although when subjects were classified into high- and low-folate groups based on the median intake, the *MTHFR* 677TT genotype conferred an increased risk regardless of folate intake. There were no significant interactions between *MTHFR* C677T genotype and vitamin B_2_, B_6_, and B_12_ intakes.

*TSER* polymorphism genotype did not significantly alter CRA risk, and further stratification into putative high and low TS expression groups on the basis of *TSER* 3R G>C polymorphism genotype did not result in a significant association (Table 2). When stratified by alcohol or nutrient intakes, from either food alone or food and supplements combined, no consistent patterns were observed. A trend towards a reduced CRA risk was observed in *MTHFR* 1298CC homozygotes, while *MTR* A2756G and *MTRR* A66G genotypes did not influence risk (Table 2), and there were no significant gene–nutrient interactions.

Linkage disequilibrium was observed between both the *TSER* and *TS* 1494del6, and the *MTHFR* C677T and A1298C polymorphisms with *r*^2^ values of 0.11 and 0.22, respectively. Four *TS* haplotypes and three *MTHFR* haplotypes were observed (Table 4). Prior functional data suggest that the *TS* 3R/ins and *MTHFR* 677A/1298C haplotypes will confer the highest TS protein expression/mRNA stability and MTHFR enzyme function, respectively (Frosst *et al*, 1995; Weisberg *et al*, 1998; Kawakami and Watanabe, 2003; Mandola *et al*, 2003, 2004). Using these haplotypes as the reference groups in haplotype analyses, no significant associations between either *MTHFR* or *TS* haplotypes and CRA risk were observed.

## DISCUSSION

Our finding of a reduced CRA risk in individuals homozygous for the *TS* 1494del6 variant adds to the increasing evidence supporting a role for altered TS expression in colorectal carcinogenesis. Presence of the deleted allele has been reported to result in enhanced TS mRNA degradation *in vitro*, and two clinical studies have observed reduced TS mRNA expression in colorectal tumours of del/del homozygote patients (Lenz *et al*, 2002; Mandola *et al*, 2004). In a recent study of CRC patients receiving 5-fluorouracil (5-FU)-based adjuvant chemotherapy, patients heterozygous or homozygous for the deleted allele had an improved survival indicating likely increased sensitivity to TS inhibition, providing further evidence that this polymorphism is functional (Dotor *et al*, 2006). Furthermore, the largest previous case–control study investigating the association of *TS* 1494del6 genotype and colorectal neoplasia risk, reported a reduced CRC risk in women homozygous for the deleted allele (Ulrich *et al*, 2005).

Our study is the first to report the association between the combined *TSER* and *TSER* 3R G>C polymorphism genotypes and colorectal neoplasia risk. Functional data from *in vitro* experiments suggest that 3RG alleles confer increased TS mRNA expression compared to either 3RC or 2R alleles, and consideration of the G>C polymorphism may allow more accurate stratification of individuals into high and low TS expression groups (Kawakami and Watanabe, 2003; Mandola *et al*, 2003). On this basis, our findings of nonsignificantly increased CRA risks in low-expression genotype groups, when categorised either by *TSER* genotype alone or in combination with the 3R G/C polymorphism, is seemingly at odds with the reduced CRA risk in individuals homozygous for the *TS* 1494del6 variant. More recent data, however, indicate that functional predictions based on *in vitro* tests may not reflect the real situation *in vivo* (Etienne *et al*, 2002; Jakobsen *et al*, 2005; Dotor *et al*, 2006). For example, while studies have reported increased TS mRNA expression in human colorectal tumours from individuals with 3R/3R genotype, the only study of TS protein activity reported an increased activity in tumours harbouring the 3R/2R genotype, indicating the existence of additional post-transcriptional regulatory pathways (Etienne *et al*, 2002). Additionally, two recent studies of 5-FU chemotherapy in patients with CRC, one in the adjuvant setting and one in patients with metastatic disease, both reported significantly improved outcomes in individuals with 3R/3R genotype (Jakobsen *et al*, 2005; Dotor *et al*, 2006). Thus prediction of TS protein activity in either normal or tumour tissue based on *TS* polymorphism genotypes is far from straightforward, and discrepancies in associations between colorectal neoplasia risk and *TS* genotypes with apparently similar functional effects are not uncommon (Ulrich *et al*, 2002, 2005; Adleff *et al*, 2004). Three previous studies, two in US populations and one in a Dutch population, have investigated the relationship between *TSER* genotype and CRA risk (without consideration of the *TSER* 3R G/C polymorphism), and in concordance with our study, all three reported no significant association (Ulrich *et al*, 2002; Chen *et al*, 2004; van den Donk *et al*, 2007). One of these studies also genotyped the *TS* 1494del6 variant and in contrast to our study found no significant influence on CRA risk (Ulrich *et al*, 2002). This discrepancy may reflect a genuine difference in the gene–disease association between different populations, effect modification due to different population intakes of micronutrients, or may simply be due to chance.

Our finding of significant interactions between folate, and vitamin B_6_ and B_12_ intakes and *TS* 1494del6 genotype provide further evidence that assessment of genotype alone will be insufficient to determine an individuals' risk of colorectal neoplasia. Similar gene–nutrient interactions have been reported with other polymorphisms in folate metabolism genes, in particular the *MTHFR* C677T variant (Ma *et al*, 1997), and it is entirely plausible that the risks conferred by low penetrance colorectal susceptibility alleles may be influenced by environmental exposures that impact on the same metabolic pathways. The reduced CRA risk associated with the homozygous del/del genotype seen in our study is most likely due to an increased availability of 5,10-methyleneTHF for use in DNA methylation. Enhancement of this protective effect by dietary factors that also result in increased 5,10-methyleneTHF, such as vitamin B_6_ and folate, and others that allow increased cycling of methyl groups towards methylation, such as vitamin B_12_, provides further evidence for this model. As does the observation that the protective effect of the del/del genotype was confined to carriers of the *MTHFR* 677CC genotype that allows efficient metabolism of 5,10-methyleneTHF.

Controls subjects in our study were recruited after the results of their colonoscopy were known, which might have led to ascertainment bias, and all subjects completed FFQs once their disease status was known, which might have led to recall bias. The use of controls known to be adenoma free, however, has the advantage of avoiding the loss of power associated with incorrect classification of cases and controls, and the indications for colonoscopy were not related to genotype for any of the variants studied. Other strengths of this study include its relatively large sample size, and the collection of data on multiple genetic and dietary factors, allowing investigation of, and where appropriate, adjustment for, gene–gene and gene–nutrient interactions. All control subjects in this study were recruited from the Midlands region, whereas 35% of cases were from non-Midlands UK areas. This difference in geographical location occurred because a proportion of case subjects recruited through UKCAP were from northern and western UK regions. Although this discrepancy has the potential to bias associations between dietary factors and CRA risk due to regional differences in diet, it will not confound genotype associations since geographical location is unlikely to be a determinant of genotype among UK Caucasians (The Wellcome Trust Case Control Consortium, 2007). In analyses restricted to cases from the Midlands region, individuals homozygous for the *TS* 1494del6 variant remained at reduced CRA risk (OR 0.50; 95% CI: 0.28–0.89), and the interactions between *TS* 1494del6 genotype and dietary intakes of folate, and vitamins B_6_ and B_12_ remained significant (*P*=0.02, 0.01, and 0.02, respectively). The majority of cases in our study were recruited from the UKCAP trial in which aspirin or NSAID use was an exclusion criterion, whereas this exclusion criterion was not applied to controls recruited to our study, making aspirin use a potential confounder. Current literature, however, provide little evidence of a relationship between aspirin or NSAID use and genotype for the variants we tested, or for interactions between genotype and aspirin or NSAID use in determining risk of CRA or CRC, hence, we feel our observations are unlikely to have been influenced by such confounding.

In summary, we observed an influence of *TS* 1494del6 genotype on CRA risk, which was largely confined to subgroups of the population defined by dietary intakes of folate and other B vitamins, and *MTHFR* C677T genotype. Our study adds to the growing body of evidence indicating that functional polymorphisms in folate metabolism genes act as low penetrance susceptibility alleles for colorectal neoplasia, and participate in complex gene–gene and gene–nutrient interactions.

## Figures and Tables

**Figure 1:**
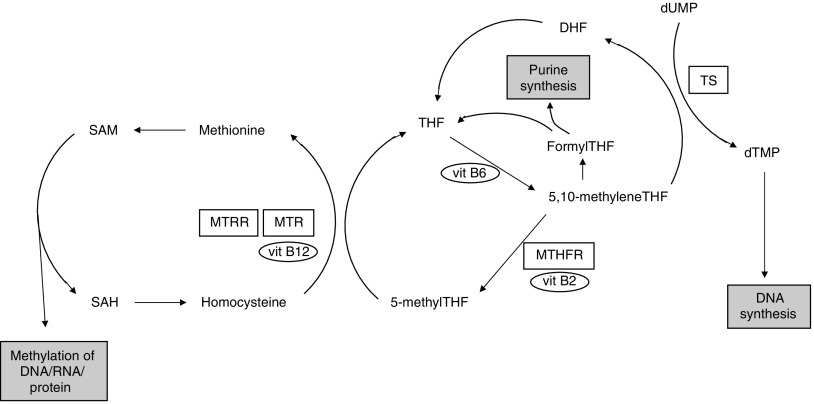
Schematic representation of folate metabolism. Corresponding enzymes for genes with polymorphisms investigated in this study are boxed. B vitamin cofactors are in ovals. TS, thymidylate synthase; MTHFR, methylenetetrahydrofolate reductase; MTR, methionine synthase; MTRR methionine synthase reductase; THF, tetrahydrofolate; DHF, dihydrofolate; dUMP, deoxyuridine monophosphate; dTMP, deoxythymidine monophoshate; SAM, *S*-adenosylmethionine; SAH, *S*-adenosylhomocysteine.

**Table 1:** Characteristics of the study population

**Variable**	**Cases (*n*=673)**	**Controls (*n*=301)**	***P* a**
*Indication for colonscopy*			
Asymptomatic/screening	6%	15%	
Bleeding/anaemia	55%	45%	
Altered bowel habit	5%	15%	
Abdominal symptoms	10%	14%	
Other	8%	19%	
Unknown	16%	6%	
			
*Location of largest adenoma*			
Proximal colon	9%		
Distal colon	64%		
Rectum	23%		
Unknown	3%		
Multiple polyps^b^	17%		
Advanced adenoma^c^	61%		
			
*Sex*			
Female	46.5%	60.5%	<0.01
Male	53.5%	39.5%	
Age^d^	57.8 (9.9)	58.6 (12.7)	0.29
Family history^e^	25%	26%	0.80
BMI^d^	26.3 (4.1)	26.3 (4.7)	0.86
			
*Smoking*			
Pack years^d^	15.6 (18.7)	12.3 (16.8)	<0.01
Never	37%	45%	
Ever	63%	55%	0.02
Regular aspirin/NSAID use^f^	28%	35%	0.22
			
*Alcohol*			
*Men*			
Mean (s. d.)^g^	19.2 (19.8)	15.9 (21.8)	0.13
None	13%	19%	
			
*Women*			
Mean (std dev)^g^	7.4 (11.9)	5.1 (6.8)	0.02
None	32%	38%	
Dietary fibre intake (g)d,^h^	20.5 (6.1)	22.4 (6.9)	<0.01
			
*Nutrient intake (excluding supplements)* d, h			
Folate (*μ*g day^−1^)	304 (96)	340 (109)	<0.01
Vitamin B_2_ (mg day^−1^)	2.47 (0.54)	2.54 (0.56)	0.10
Vitamin B_6_ (mg day^−1^)	2.25 (0.56)	2.46 (0.67)	<0.01
Vitamin B_12_ (*μ*g day^−1^)	6.80 (3.04)	6.92 (3.01)	0.58
			
*Nutrient intake (including supplements)* d, h			
Folate (*μ*g day^−1^)	336 (129)	387 (152)	<0.01
Vitamin B_2_ (mg day^−1^)	2.70 (0.83)	2.87 (0.96)	0.01
Vitamin B_6_ (mg day^−1^)	2.54 (0.94)	2.88 (1.20)	<0.01
Vitamin B_12_ (*μ*g day^−1^)	6.95 (3.07)	7.13 (3.05)	0.41

BMI, body mass index.

a *P*-value based on *χ*^2^ and *t*-tests.

b ⩾3 polyps.

c Villous or tubulovillous features, size ⩾1 cm, or severe dysplasia.

d Mean (s.d.).

e Family history of colorectal cancer in one or more first-degree relative.

f At least one tablet per month for at least a year, data for cases includes only those recruited outside the UKCAP trial (*n*=107), since aspirin or NSAID use was an exclusion criterion for this trial.

g Units per week.

h Energy-adjusted dietary nutrient intakes.

**Table 2:** Association of genotype and colorectal adenoma risk

**Variant**	**Genotype**	**No. of case/control**	**Unadjusted OR (95% CI)**	**Adjusted OR (95% CI)^a^**
*TSER*	3R/3R	191/101	Ref	Ref
	3R/2R	330/133	1.31 (0.96–1.80)	1.21 (0.86–1.69)
	2R/2R	147/66	1.18 (0.81–1.72)	1.19 (0.79–1.80)
				
*TSER* High/low^b^	High	257/126	Ref	Ref
	Low	411/174	1.16 (0.88–1.53)	1.16 (0.86–1.56)
				
*TS* 1494del6	Ins/ins	337/143	Ref	Ref
	Ins/del	288/119	1.03 (0.77–1.37)	0.97 (0.71–1.33)
	Del/del	48/39	0.52 (0.33–0.83)†	0.51 (0.30–0.85)†
				
*MTHFR* C677T	CC	306/143	Ref	Ref
	CT	289/133	1.02 (0.76–1.35)	0.99 (0.73–1.35)
	TT	78/25	1.46 (0.89–2.39)	1.71 (1.00–2.92)‡
				
*MTHFR* A1298C	AA	331/135	Ref	Ref
	AC	277/128	0.88 (0.66–1.18)	0.85 (0.62–1.16)
	CC	65/38	0.70 (0.45–1.09)	0.65 (0.41–1.05)
				
*MTR* A2756G	AA	461/191	Ref	Ref
	AG	191/98	0.81 (0.60–1.09)	0.80 (0.57–1.09)
	GG	21/12	0.73 (0.35–1.50)	0.99 (0.45–2.15)
				
*MTRR* A66G	AA	140/58	Ref	Ref
	AG	322/137	0.97 (0.68–1.40)	0.98 (0.66–1.45)
	GG	211/106	0.82 (0.56–1.21)	0.86 (0.57–1.30)

OR=Odds ratio; 95% CI=95% confidence interval.

a Adjusted for sex, smoking, alcohol consumption, and dietary fibre, folate, and vitamins B_2_ and B_6_ (from food and supplements).

b High-expression *TSER* genotypes are 3RG/3RG, 3RG/3RC, and 3RG/2RG, and low expression genotypes are 3RC/3RC, 3RC/2RG, and 2RG/2RG.

† *P*=0.01

‡ *P*=0.05.

**Table 3:** Association of *TS* 1494del6 and *MTHFR* C677T genotypes and colorectal adenoma risk stratified by nutrient intake

	**Genotype**			
	***TS*1494del6**			
**Tertiles of nutrient intake from food only^a^**	**Ins/ins**	**Ins/del**	**Del/del**	***P*_interaction_**
*Folate*				
*High*				
No. of cases/controls	63/54	49/23	5/14	
OR (95% CI)^b^	Ref	1.82 (0.96–3.49)	0.30 (0.10–0.95)	
				
*Low/medium*				
No. of cases/controls	240/81	189/85	37/20	
OR (95% CI)^b^	1.53 (0.89–2.64)	1.13 (0.65–1.95)	1.19 (0.51–2.75)	0.01
				
*Vitamin B_2_*				
*High*				
No. of cases/controls	84/49	52/33	7/10	
OR (95% CI)^b^	Ref	1.00 (0.56–1.80)	0.35 (0.12–1.01)	
				
*Low/medium*				
No. of cases/controls	219/86	186/75	35/24	
OR (95% CI)^b^	1.05 (0.66–1.69)	1.07 (0.66–1.73)	0.55 (0.26–1.17)	0.86
				
*Vitamin B_6_*				
*High*				
No. of cases/controls	59/53	57/25	8/13	
OR (95% CI)^b^	Ref	2.26 (1.21–4.23)	0.52 (0.20–1.41)	
				
*Low/medium*				
No. of cases/controls	244/82	181/83	34/21	
OR (95% CI)^b^	1.54 (0.90–2.65)	1.09 (0.61–1.94)	1.46 (0.46–4.59)	0.006
				
*Vitamin B_12_*				
*High*				
No. of cases/controls	87/43	61/29	9/19	
OR (95% CI)^b^	Ref	1.23 (0.67–2.29)	0.25 (0.10–0.63)	
				
*Low/medium*				
No. of cases/controls	216/92	177/79	33/15	
OR (95% CI)^b^	1.39 (0.86–2.24)	1.00 (0.62–1.62)	1.00 (0.45–2.22)	0.03
				
*Alcohol*				
*Low*				
No. of cases/controls	94/40	77/47	8/13	
OR (95% CI)^b^	Ref	0.61 (0.35–1.05)	0.23 (0.08–0.71)	
				
*High/medium*				
No. of cases/controls	243/103	211/72	40/26	
OR (95%CI)^b^	0.89 (0.55–1.42)	1.04 (0.63–1.73)	0.57 (0.28–1.14)	0.11
*Folate*				
*High*				
No. of cases/controls	51/37	47/46	19/8	
OR (95% CI)^b^	Ref	0.71 (0.38–1.34)	1.79 (0.67–4.78)	
				
*Low/medium*				
No. of cases/controls	214/93	201/79	51/14	0.59
OR (95% CI)^b^	1.44 (0.81–2.57)	1.37 (0.76–2.48)	2.28 (0.94–5.57)	
				
*Alcohol*				
*Low*				
No. of cases/controls	81/53	79/40	19/7	
OR (95% CI)^b^	Ref	1.29 (0.74–2.24)	1.67 (0.63–4.46)	
				
*High/medium*				
No. of cases/controls	225/90	210/93	59/18	
OR (95% CI)^b^	1.47 (0.92–2.33)	1.30 (0.80–2.09)	2.41 (1.21–4.83)	0.44

a Cut points for tertiles of dietary intakes: folate (*μ*g day^−1^) 295/383; vitamin B_2_ (mg day^−1^) 2.29/2.84; vitamin B_6_ (mg day^−1^) 2.18/2.71; vitamin B_12_ (*μ*g day^−1^) 5.30/7.65, alcohol (units per week) men 3/15, women 1/8.

b Odds ratios and 95% confidence intervals adjusted for sex, smoking, alcohol consumption, and dietary intakes of fibre, folate, and vitamins B_2_ and B_6_ (from food and supplements) where appropriate.

**Table 4:** Association of *TS* and *MTHFR* haplotypes and colorectal adenoma risk

**Gene**	**Haplotype**	**Frequency (%)**	**No. of case/control**	**OR (95% CI)^a^**
TS	3R/ins	28.6	384/169	Ref
	2R/ins	41.7	572/236	1.02 (0.79–1.31)
	3R/del	25.5	328/166	0.80 (0.61–1.07)
	2R/del	4.2	52/29	0.84 (0.49–1.45)
MTHFR	677C/1298A	36.4	494/215	Ref
	677T/1298A	32.2	445/183	0.92 (0.66–1.29)
	677C/1298C	31.4	407/204	0.85 (0.66–1.09)

a Odds ratios and 95% confidence intervals adjusted for sex, smoking, alcohol consumption, and dietary intakes of fibre, folate, and vitamins B_2_ and B_6_ (from food and supplements).
